# Ganoderma lucidum polysaccharide attenuates retinal ischemia-reperfusion injury by regulating microglial M1/M2 polarization, suppressing neuroinflammation and inhibiting JAK2/STAT3 pathway

**DOI:** 10.1016/j.bbrep.2025.101926

**Published:** 2025-01-29

**Authors:** Guangyu Zhu, Yujie Liu, Shichun Luo, Chao Tang, Chunlin Zhao, Xuejing Lu

**Affiliations:** a Eye School of Chengdu University of TCM, Chengdu, 610075, China; b Ineye Hospital of Chengdu University of TCM, Chengdu, 610075, China; c Key Laboratory of Sichuan Province Ophthalmopathy Prevention & Cure and Visual Function Protection with TCM Laboratory, Chengdu, 610075, China; d Retinal Image Technology and Chronic Vascular Disease Prevention & Control and Collaborative Innovation Center, Chengdu, 610075, China

**Keywords:** Neuroinflammation, Microglia, Ganoderma lucidum polysaccharide, Retinal ischemia-reperfusion, JAK2/STAT3 signaling pathway

## Abstract

Retinal ischemia-reperfusion (RIR) injury is implicated in the pathogenesis of numerous retinal degenerative disorders, resulting in visual impairment or even blindness in millions of individuals worldwide. In recent years, targeting the suppression of microglia-mediated neuroinflammation has emerged as a principal therapeutic strategy for RIR. Ganoderma lucidum polysaccharide (GLP), a pivotal bioactive extract of Ganoderma lucidum, has been demonstrated to possess efficacious anti-neuroinflammatory properties, while the precise impact of it on RIR injury remains incompletely elucidated. In this study, the RIR model was induced in Sprague-Dawley rats by elevating the intraocular pressure to 80 mmHg for 60 min. Our findings revealed that GLP significantly alleviated inflammatory processes by impeding the secretion of pro-inflammatory cytokines while facilitating that of anti-inflammatory cytokines. Moreover, the administration of GLP also promoted the polarization of microglia from the M1 phenotype to the M2 phenotype. Further investigation of the treatment mechanism indicated that the regulatory effects of GLP were presumably mediated by the inhibition of the JAK2/STAT3 signaling pathway. To summarize, we provided a novel insight into the mechanisms by which GLP ameliorated RIR injury, thereby indicating that it could be identified as a promising candidate for the management of RIR-related diseases.

## Highlights

• GLP protects RGCs after retinal ischemia-reperfusion injury.
• GLP suppresses microglia-mediated inflammation.
• JAK2/STAT3 is involved in neuroinflammation and switch of microglial phenotype.

## 1 Introduction

Ischemia-reperfusion injury, a common pathological state initiated with a restriction of blood supply resulting in shortages of oxygen and glucose followed by subsequent reperfusion, is associated with a wide spectrum of degenerative diseases [1,2]. Characterized by irreversible loss of retinal ganglion cells (RGCs), retinal ischemia-reperfusion injury (RIR) is a hallmark of various vision-threatening disorders, including glaucoma, diabetic retinopathy, and central retinal artery/vein occlusion [3]. This process involves a complex interplay of inflammation, mitochondrial dysfunction, calcium overload, and free radical production [4,5]. Due to the complexity of these mechanisms, effective therapy targeting RIR has yet to be identified.

Increasing evidence has confirmed that neuroinflammation, which involves activation and mobilization of microglia, infiltration of granulocytes, and accumulation of monocyte/macrophages, is one of the most common cellular events following RIR injury [6]. Prolonged or excessive neuroinflammatory response is detrimental, inhibiting axonal regeneration as well as aggravating neurological dysfunction [7]. Microglia, resident macrophages in the central nervous system (CNS), are presumed to be the key mediators of neuroinflammation in many neurodegenerative diseases like glaucoma [8,9]. When RIR injury occurs, it can be polarized into two distinct phenotypes, namely M1 phenotype (pro-inflammatory) and M2 phenotype (anti-inflammatory), depending on their morphology and function [10,11]. Studies have demonstrated that the M1 phenotype, expressing surface markers such as CD86 and inducible nitric oxide synthase (iNOS), secretes pro-inflammatory cytokines and proteases including tumor necrosis factor-α (TNF-α), matrix metalloproteinase-9 (MMP-9), and interleukin-1β (IL-1β). In contrast, the M2 phenotype, characterized by CD206 and arginase-1 (Arg-1), produces diverse factors such as interleukin-4 (IL-4), brain-derived neurotrophic factor (BDNF), and interleukin-10 (IL-10) [12,13]. Acute inflammatory responses and cytotoxicity were promoted by M1 microglia while M2 microglia mainly participate in inflammation resolution, tissue repair, and phagocytosis of debris. Switching between these two phenotypes may therefore be associated with neuroinflammation induced by RIR [[14], [15], [16]]. Furthermore, the activation of the Janus Kinase 2 (JAK2)/signal transducer and activator of transcription 3 (STAT3) signaling pathway, possessing the capability to uphold the reactivity of glial cells, is indispensable for neuroinflammation mediated by microglia and astrocytes [17,18].

Ganoderma lucidum, also known as “Lingzhi” or “Reishi”, has been widely used as a natural medicine and dietary supplement in East Asia for over 2000 years, especially in China [19]. It possesses more than 400 bioactive compounds, including polysaccharides, triterpenoids, polyphenols, sterols, and nucleotides [20]. As a major extract of “Lingzhi”, Ganoderma lucidum polysaccharide (GLP) has exhibited its anti-inflammatory [21], antioxidant [22], immunoregulatory [23], and hypoglycemic properties [24]. Multiple studies have demonstrated that GLP exerted potent anti-inflammatory effects across a range of diseases and possessed neuroprotective properties following CNS injury [[25], [26], [27], [28], [29]]. For instance, it has been reported that pre-treatment with GLP could attenuate LPS-induced acute pneumonia in mice by suppressing the infiltration of inflammatory cells and the secretion of cytokines [25]. Another study indicated that the impact of GLP-1, a galactoglucan extracted from the fruiting bodies of G. lucidum, on ameliorating cognitive impairment is mediated through the modulation of inflammation via the brain-liver axis [26]. Besides that, by regulating inflammation-mediated microglial migration, morphological transformations, and behavioral responses stimulated by LPS or Aβ, GLP exerted neuroprotective effects both in vitro and in vivo [27]. The application of GLP in inhibiting neutrophil extracellular traps and the production of inflammatory cytokines has also been found to effectively attenuate ischemia-reperfusion injury in intestinal and spinal cord tissues, respectively [28,29]. Nevertheless, no studies assessing the impacts of GLP on RIR have been reported.

Currently, the precise effects and mechanisms underlying the modulation of neuroinflammatory responses and microglial M1/M2 polarization by GLP after RIR have not yet been elucidated. Based on the evidence above, we hypothesized that GLP could modulate the microglial phenotype and suppress the inflammatory responses by inhibiting the JAK2/STAT3 signaling pathway, thereby alleviating neuroinflammatory injury of retinal tissues caused by RIR.

## 2 Material and methods

### 2.1 Animals

The study was performed using 72 adult female Sprague-Dawley (SD) rats (aged 8 weeks and weight: 200 ± 20 g) obtained from Chengdu Dossy Experimental Animals Company (License No. SCXK 2020–030). These rats were housed in the Key Laboratory of Sichuan Province Ophthalmopathy Prevention & Cure and Visual Function Protection with TCM Laboratory (Chengdu, China) under standardized conditions with a temperature of 22 ± 2 °C, humidity of 55 ± 5 %, a 12-h light/dark cycle, and free access to food and water. All animal experiments complied with the ARRIVE guidelines and were conducted according to the Association for Research in Vision and Ophthalmology Statement for the Use of Animals in Ophthalmic and Vision Research. Procedures involving animals were approved by the Committee of laboratory Animal Welfare Ethics of Chengdu University of Traditional Chinese Medicine (SYXK 2019–0049). The rats were randomized into the following groups: the control group (n = 12), the RIR group (n = 12), the RIR+35 mg/kg GLP group (n = 12), the RIR+70 mg/kg GLP group (n = 12), and the RIR+140 mg/kg GLP group (n = 12). GLP, the purity of which attained 98 %, was bought from Shaanxi Jinchi Plant Technology Co., Ltd.

### 2.2 RIR model

The RIR model was established as previously reported, where the animals were primarily anesthetized with 2 % sodium pentobarbital (0.2 ml/100 g, i. p.), 1 % tropicamide, and 0.5 % proparacaine hydrochloride was applied for pupil dilation and topical anesthesia, respectively. After sterile preparation, the anterior chamber was cannulated obliquely using a 30-gauge needle, which was attached to a reservoir filled with 0.9 % saline solution via a plastic tube. To achieve an intraocular pressure (IOP) of roughly 80 mmHg, this reservoir was raised above the level of the eyeball (130 cm) for 60 min [30,31]. Completion of retinal ischemia was confirmed by observing blanching of the fundus by microscopic examination. Then the needle was removed from the anterior chamber, after which the presence of reperfusion was judged by the orange-red color of the retina by detecting the fundus again. Body temperature was maintained at 37 °C using a heating blanket throughout the procedure, and ofloxacin gel was applied to the conjunctival sac for infection prevention. While in the control group, the cornea was cannulated in the same way without the saline reservoir being raised. In the next 7 days, rats received GLP or saline solution once a day intragastrically until being sacrificed by 2 % sodium pentobarbital (1.2 ml/100 g) for further studies (Fig. 1A).

**Fig. 1:**
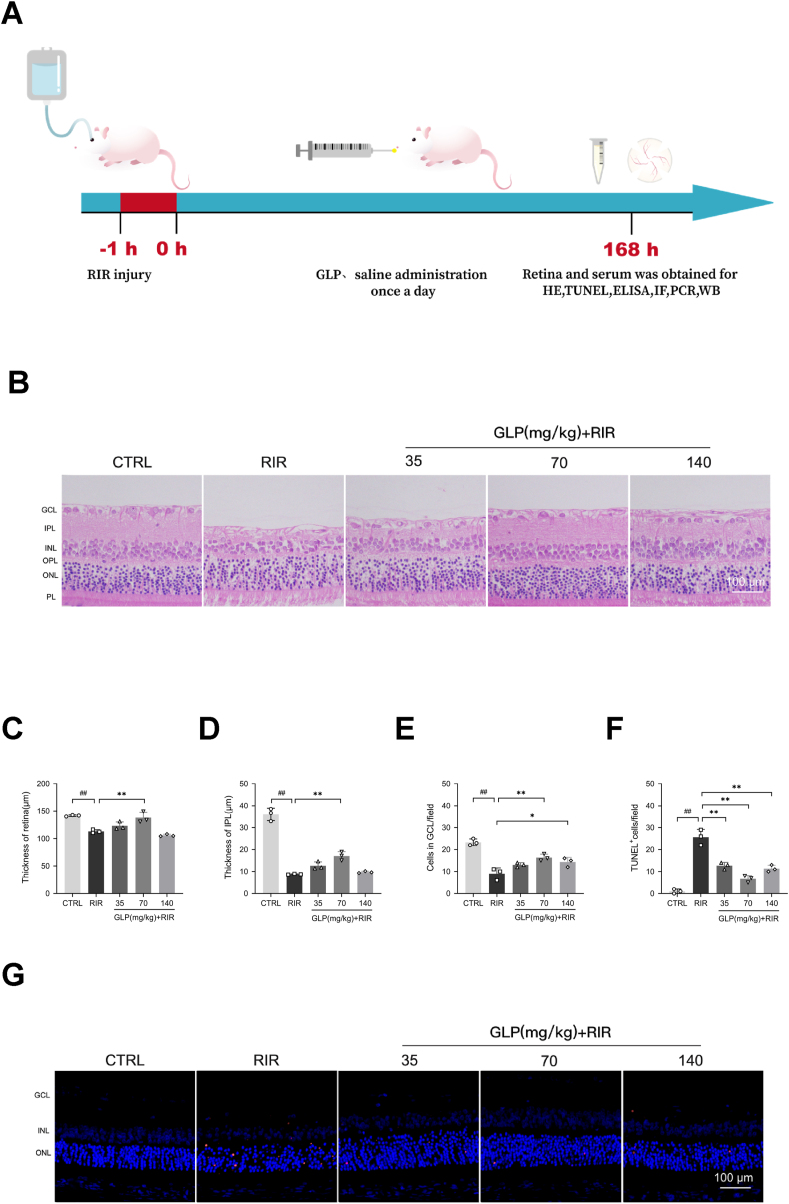
GLP exerted neuroprotective effects within the retina caused by RIR. (A) Experimental schedule for the administration of GLP to alleviate RIR injury. (B) Representative HE staining images of retinal sections in each group. Scale bar = 100 μm; GCL, ganglion cell layer; IPL, inner plexiform layer; INL, inner nuclear layer; OPL, outer plexiform layer; ONL, outer nuclear layer; PL, photoreceptor layer. (C–E) Retinas exposed to RIR injury showed that the thickness of both retina and IPL was decreased as well as significant RGC loss. (F–G) Representative TUNEL staining images, TUNEL^+^ cells (apoptotic cells) were stained red. The results showed that GLP administration significantly reduced the number of TUNEL^+^ cells. Scale bar = 100 μm; All data were presented as mean ± SD (n = 3); #p < 0.05, ##p < 0.01 compared with the control group, ∗p < 0.05, ∗∗p < 0.01 compared with the RIR group.

### 2.3 Hematoxylin and eosin (HE) staining

The eyeballs were obtained and fixed with 4 % paraformaldehyde for 24 h at 4 °C, after which the tissue was embedded in paraffin and sectioned into 5-μm-thick sections. The paraffin sections were immersed in sequence in dewaxing transparent liquid, anhydrous ethanol, and 75 % Ethyl alcohol. After being stained with hematoxylin solution and eosin dye, the sections were placed into absolute ethanol, normal butanol, and xylene and sealed with neutral gum. The thickness of the retina, from the ganglion cell layer (GCL) to the photoreceptor layer (PL) and each layer of the retina, was inspected and analyzed in four cross-sectional areas (within 1 mm from the optic nerve) using a microscope (400 × , NIKON, Japan).

### 2.4 Terminal deoxynucleotidyl transferase dUTP nick end labeling (TUNEL) staining

Qualitative analysis of neuronal apoptosis was implemented using a TUNEL assay kit (Servicebio, Chengdu, China) according to the manufacturer's instructions. Following conventional dewaxing with xylene, pure ethanol, and water, the slices were treated with proteinase K (10 μg/ml in PBS) for 20 min, permeabilized with PBS containing 0.1 % Triton for 20 min, and treated with equilibrium buffer at room temperature. Afterward, the sections were added with TdT reaction solution (TDT enzyme, dUTP, and buffer, 1:5:50) in a moist chamber at 37 °C for 1 h and rinsed before DAPI solution and anti-fade mounting medium were dripped for counterstaining and sealing, respectively. TUNEL-positive cells stained with DAPI were visualized and counted under Ortho-Fluorescent Microscopy (400 × , NIKON, Japan) in 3 randomly chosen fields.

### 2.5 Immunofluorescence (IF)

The cell status was detected by IF. Following dewaxing and antigen repair, the slices were blocked in 3 % bovine serum albumin for 30 min, incubated overnight at 4 °C with two primary antibodies mixed already, and then incubated with secondary antibodies at room temperature for 50 min in the dark. The sections were subsequently rinsed before DAPI solution, autofluorescence quencher B solution, and anti-fade mounting medium were added. The nuclei stained with DAPI were detected and counted under Ortho-Fluorescent Microscopy (400 × , NIKON, Japan) in 3 randomly chosen fields.

### 2.6 Enzyme-linked immunosorbent assay (ELISA)

The serum samples were obtained from the rats' orbits, which were then placed at room temperature for 2 h. The supernatant was collected by centrifugation at 3000*g* at 4 °C for 15 min, and the protein concentration was determined using a bicinchoninic acid assay kit (G2026-200T, Servicebio, Chengdu, China). Commercially available ELISA kits for measuring the levels of TNF-α (GER0004, Servicebio, Chengdu, China), IL-1β (GER0002, Servicebio, Chengdu, China), IL-10 (GER0003, Servicebio, Chengdu, China), and IL-6 (GER0001, Servicebio, Chengdu, China) according to the manufacturer's instructions. Optical density at 450 nm was determined using an Enzyme label detector (Epoch; BioTek Instruments, Inc.)

### 2.7 Quantitative real-time polymerase chain reaction (RT-qPCR)

Total RNA was extracted from retinal tissues and quantified with Nanodrop 2000 (Thermo Scientific, Wilmington, DE, USA). The first-strand cDNA was synthesized with SweScript All-in-One SuperMix for qPCR (Servicebio, Chengdu, China), followed by the use of RT-qPCR System (Bio-Rad Laboratories, Inc.) with Universal Blue SYBR Green qPCR Master Mix according to the manufacturer's instructions. The Delta CT method was used to calculate the expression of the gene, the results of which were normalized by GAPDH. All primers were synthesized by Wuhan Service Technology Co., Ltd., and forward and reverse primers used were as follows:

CD86 (f: 5’-GTGTTTGAAGATGCAGAACCAGC-3’, r: 5’-GCAATTGGGGTTCAACTTCCTC-3’); CD206 (f: 5’-CATCTGCCAGCGACATAATAGC -3’, r: 5’-GGACACCAGGTTTCCTTTCAATC-3’); GAPDH (f: 5’-CTGGAGAAACCTGCCAAGTATG -3’, r: 5’-GGTGGAAGAATGGGAGTTGCT-3’)

### 2.8 Western Blot (WB)

The total proteins from the tissues were extracted after homogenization with RIPA buffer (Servicebio, Chengdu, China) containing protease inhibitors and phosphatase inhibitors (RIPA: protease inhibitors: phosphatase inhibitors = 100:1:1, Servicebio, Chengdu, China). After centrifugation at 12,000 rpm for 10 min at 4 °C, the supernatants were collected, which were then measured using a BCA kit (Servicebio, Chengdu, China). The protein was resolved by electrophoresis on SDS-PAGE gels under 120 V and electroblotted onto PVDF membranes. Blocked with 0.1 % Tween 20 containing 5 % fat-free milk for 30 min at room temperature, the membranes were incubated with primary antibodies overnight at 4 °C. On the following day, the membrane was incubated with secondary antibodies at room temperature for 30 min after being rinsed three times with TBS-T. The membranes were visualized with ECL reagent (Servicebio, Chengdu, China) by using a chemiluminescence apparatus (Servicebio, Chengdu, China), and the bands were quantified using Image J software, GADPH being used as the internal reference. The primary antibodies used in this study were JAK2 (Servicebio, Rabbit. #GB11325), P-JAK2 (BIOSS, Rabbit. # bs-3206r), STAT3 (Servicebio, Rabbit. #GB11176), and P-STAT3 (Servicebio, Rabbit. # GB150001). Secondary antibodies (Servicebio. # GB23303) were diluted 1:3000 in 5 % milk.

### 2.9 Statistical analysis

All statistical analyses were conducted using GraphPad Prism 9 (GraphPad Software, San Diego, CA, USA), with all data presented as mean ± SD. A one-way analysis of variance was performed, followed by a Tukey post-hoc test, for comparing multiple groups. *P*-value< 0.05 was considered statistically significant.

## 3 Results

### 3.1 GLP alleviates neuronal damage within the retina caused by RIR

The therapeutic effects of GLP were investigated by establishing an RIR model in vivo, after which the rats received GLP solution intragastrically to simulate the drug delivery strategy typically employed in traditional Chinese medicine. Our investigation mainly focused on the ganglion cell complex (GCC), comprising the retinal nerve fiber layer (RNFL), GCL, and inner plexiform layer (IPL), which was identified as the primary site suffering neuronal damage upon sustaining RIR injury [32,33].

After RIR injury, the thickness of both retina and IPL were decreased in the RIR group (Fig. 1B–D), which was significantly reversed by the administration of 70 mg/kg GLP (Fig. 1B–D). Additionally, both 70 mg/kg GLP and 140 mg/kg GLP had significant impacts on maintaining the number of cells in the GCL (Fig. 1B and E).

In another aspect, TUNEL staining revealed that the administration of all three doses of GLP significantly decreased the number of apoptotic cells across the entire retina (Fig. 1F and G). These data suggested that GLP possessed neuroprotective properties following RIR injury.

### 3.2 GLP inhibits the secretion of pro-inflammatory cytokines and promotes that of anti-inflammatory cytokines following RIR

**Fig. 2:**
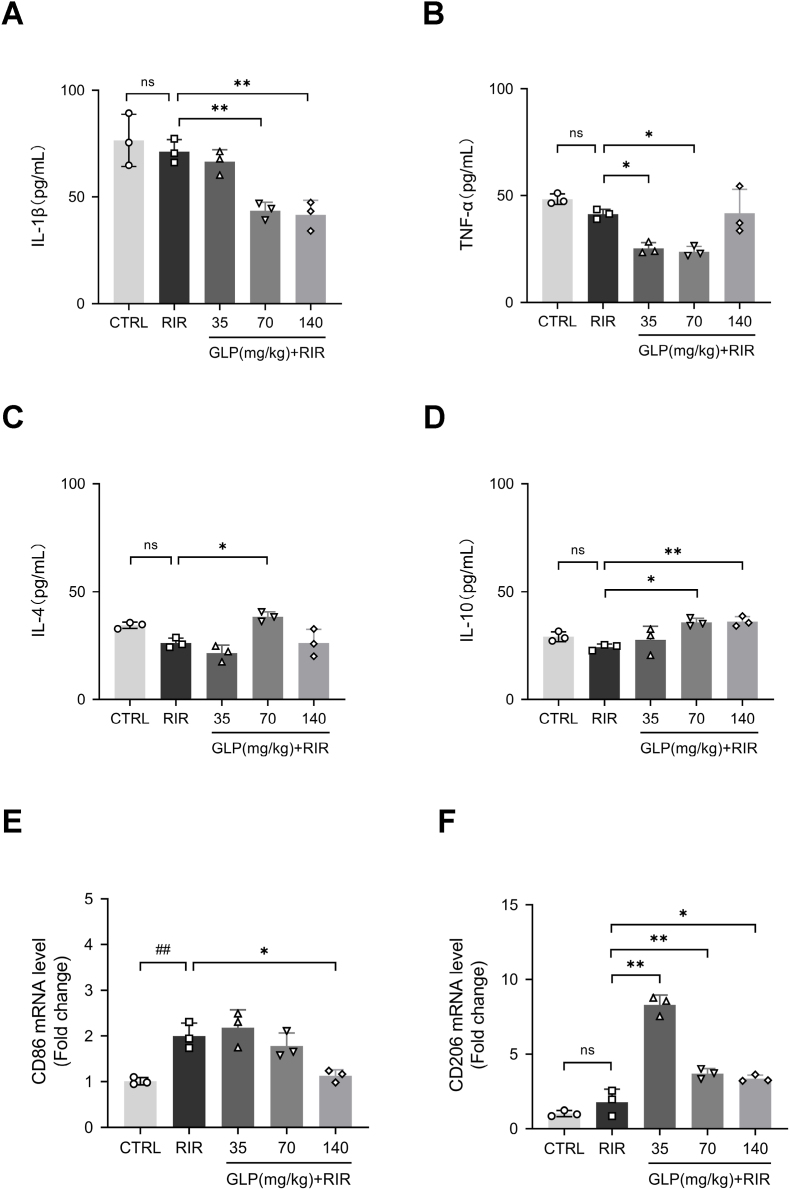

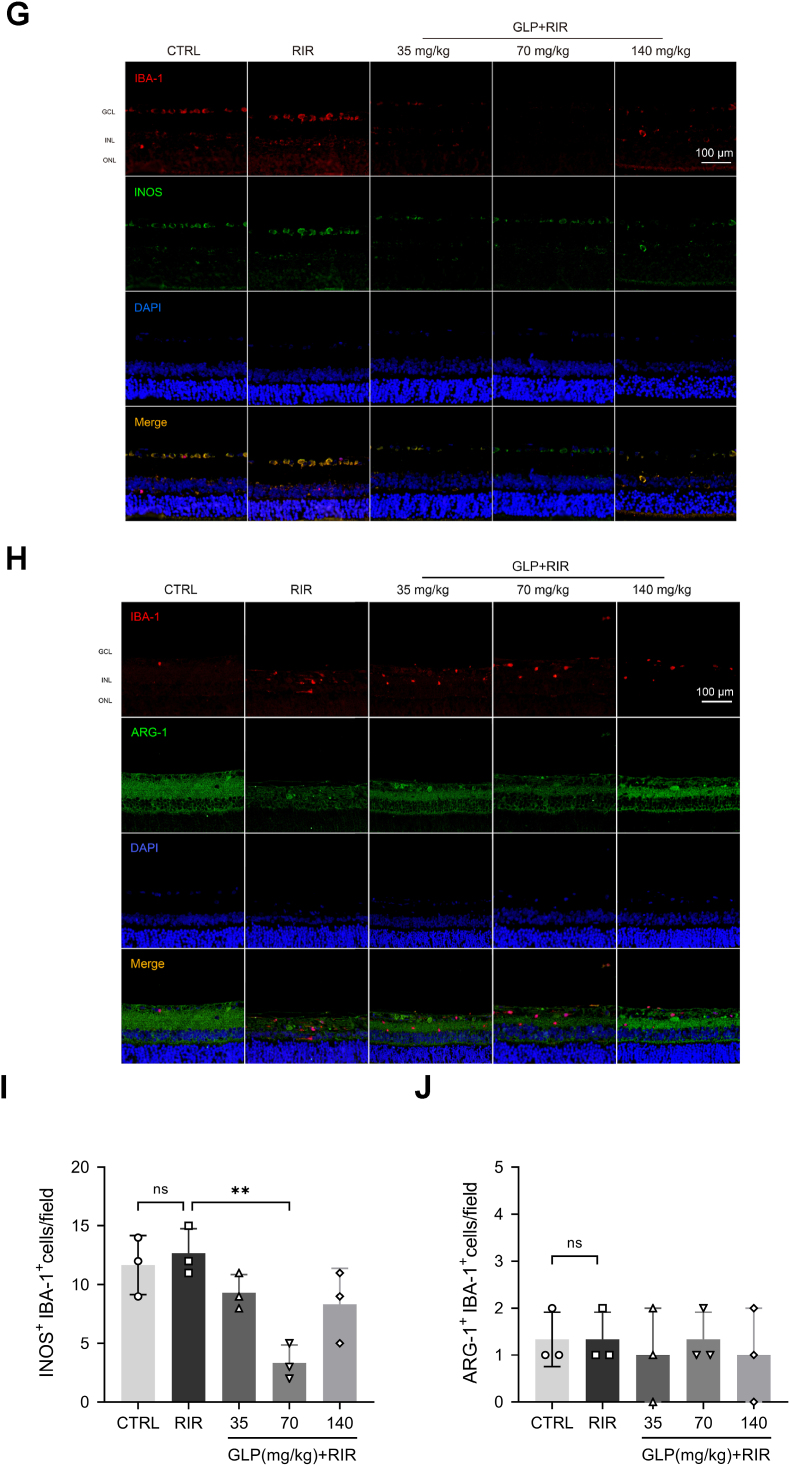
GLP suppressed the microglia-mediated inflammation following RIR. (A–D) ELISA showed that GLP reduced the concentrations of IL-1β and TNF-α while increasing the concentrations of IL-4 and IL-10 after RIR. (*E*–F) The mRNA levels of CD86 and CD206 were quantified by RT-qPCR. The results indicated that the mRNA levels of CD86 decreased in the 140 mg/kg GLP group, and the administration of all three doses of GLP increased the levels of CD206. (G–H) Representative fluorescent co-labeled staining of IBA-1 (red) with INOS (green) and IBA-1 (red) with ARG-1 (green), the co-labeled cells were stained yellow. Scale bar = 100 μm. (I–J) The number of IBA-1^+^ INOS^+^ cells and IBA-1^+^ ARG-1^+^ cells in each field. Administration of GLP significantly suppressed M1 microglial polarization (IBA-1^+^ INOS^+^ cells), while there was no significant difference among the 5 groups. All data were presented as mean ± SD (n = 3); #p < 0.05, ##p < 0.01 compared with the control group, ∗p < 0.05, ∗∗p < 0.01 compared with the RIR group.

We focused on the severity of inflammation following RIR injury by assessing the levels of inflammatory factors. The concentrations of pro-inflammatory cytokines (IL-1β, TNF-α) and anti-inflammatory cytokines (IL-4, IL-10) in rat serum were quantified by ELISA. Compared with the RIR group, the concentrations of IL-1β were decreased in the 70 mg/kg GLP and 140 mg/kg GLP groups (Fig. 2A), and those of TNF-α were reduced in the 35 mg/kg GLP and 70 mg/kg GLP groups (Fig. 2B).

With regard to anti-inflammatory cytokines, the administration of 70 mg/kg GLP up-regulated the levels of IL-4 and IL-10 (Fig. 2C and D), while 140 mg/kg GLP significantly increased the concentrations of IL-10 (Fig. 2D). While there appear to be no changes in IL-1β, TNF-α, IL-4, and IL-10 levels between the Control and RIR groups, it may be attributed to the selected time point at which the serum samples were obtained, considering that the levels of cytokine induced by RIR injury may have already returned to normal after 7 days. Nonetheless, we observed the tendency that GLP could relieve RIR-induced inflammation by inhibiting the secretion of pro-inflammatory cytokines along with promoting that of anti-inflammatory cytokines.

### 3.3 GLP inhibits the proliferation of M1 microglia and promotes that of M2 microglia following RIR

We hypothesized that GLP exerts anti-inflammatory effects by modulating the microglial phenotype. Therefore, the gene expression of the markers of M1 microglia (CD86) and M2 microglia (CD206) was determined by RT-qPCR. The results indicated that RIR injury significantly increased the mRNA levels of CD86 (Fig. 2E), which decreased in the 140 mg/kg GLP group (Fig. 2E). However, the levels of CD206 were increased in the 140 mg/kg GLP group (Fig. 2F), with a more significant elevation in the 35 mg/kg GLP and 70 mg/kg GLP groups (Fig. 2F).

To further verify it, fluorescent co-labeled staining of IBA-1 with INOS or ARG-1 was used. Fluorescence intensity analysis revealed that 70 mg/kg GLP significantly reduced the number of IBA-1^+^ iNOS^+^ cells (Fig. 2G and H), while there were no significant differences in the number of IBA-1^+^ ARG-1^+^ cells between each group (Fig. 2I). This may be attributed to the selected time point at which the retina was obtained, as quite a few investigators employed observation nodes at either 24 or 72 h [34,35]. Taken together, these findings suggested that GLP administration can suppress inflammatory responses by promoting the polarization of microglia from the M1 phenotype to the M2 phenotype.

### 3.4 GLP inhibits JAK2/STAT3 signaling pathway following RIR

**Fig. 3:**
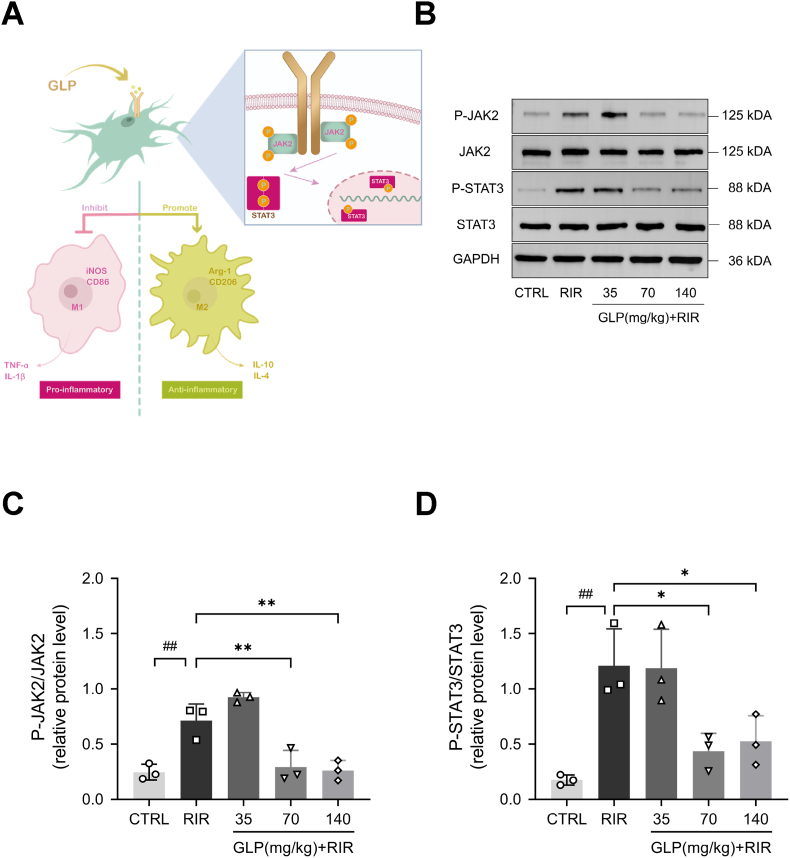
GLP regulated the JAK2/STAT3 signaling pathway in RIR injury. (A) Schematic diagram of the JAK2/STAT3 signaling pathway by which GLP suppressed the microglia-mediated inflammation. (B) Representative bands for JAK2/STAT3 pathway proteins (P-JAK2, JAK2, P-STAT3, STAT3). (C–D) The phosphorylation levels of JAK2 and STAT3 were quantified using the ratios of P-JAK2/JAK2 and P-STAT3/STAT3, the results of which indicated that GLP inhibited the JAK2/STAT3 signaling pathway. All data were presented as mean ± SD (n = 3). #p < 0.05, ##p < 0.01 compared with the control group, ∗p < 0.05, ∗∗p < 0.01 compared with the RIR group.

Previous investigations have shown that the activation of the JAK2/STAT3 signaling pathway may be closely associated with neuroinflammation mediated by microglia [36,37]. Therefore, we hypothesized that GLP exerts neuroprotective effects against RIR-induced injury by inhibiting the JAK2/STAT3 signaling pathway and, consequently, microglia-mediated neuroinflammation (Fig. 3A).

In this study, the phosphorylation levels of JAK2 and STAT3 were quantified using the ratios of P-JAK2/JAK2 and P-STAT3/STAT3, respectively. WB analyses revealed that the phosphorylation levels of JAK2 and its downstream protein STAT3 were significantly upregulated in the RIR group compared with the control group (Fig. 3B–D), which indicated that the JAK2/STAT3 signaling pathway was activated following RIR injury. However, the administration of GLP reversed this phenomenon, as evidenced by the decreased ratios of both P-JAK2/JAK2 and P-STAT3/STAT3 in retinal tissues (Fig. 3B–D). In summary, the JAK2/STAT3 signaling pathway may, at least in part, elucidate the therapeutic effects of GLP on modulating the microglial phenotype and suppressing the neuroinflammatory responses following RIR injury.

## 4 Discussion

An increasing number of researchers are exploring the potential benefits of bioactive compounds derived from medicinal fungus, given their advantages of safety and fewer side effects when compared to chemical pharmaceuticals [38]. Over the last few years, GLP, a water-soluble extract of Ganoderma lucidum, has been proven to exert potent anti-inflammatory effects and possess neuroprotective properties in several neuroinflammatory disorders, such as cognitive impairment [39], major depressive disorder [40], and spinal cord ischemia-reperfusion injury [29]. In this study, we investigated the neuroprotective properties of GLP against RIR-induced neuroinflammation by regulating microglial M1/M2 polarization, probably mediated by the JAK2/STAT3 pathway.

Recently, substantial evidence has suggested that neuroinflammation is a major contributor to the pathogenesis of RIR [[41], [42], [43]]. Neuroinflammation, implicated in the pathophysiology of various neurological disorders, is a complex innate response initiated towards ischemia, infection, and trauma by several immune cells, especially microglia [44]. Acute neuroinflammation is mostly protective and beneficial by eliminating pathogens or promoting the repair of tissues, while excessive neuroinflammation is always detrimental due to the release of pro-inflammatory cytokines and cytotoxic factors mediated by microglia [45]. Although oversimplified, the concept that microglia were classified into two opposing phenotypes: M1 (classical activation) and M2 (alternative activation) phenotypes is still widely acknowledged [46]. The M1 phenotype promotes neuronal degeneration and dysfunction of neural networks by secreting pro-inflammatory cytokines and proteases [12]; In contrast, the M2 phenotype contributes to inflammation resolution, tissue repair, and phagocytosis of debris by producing diverse factors [13]. An imbalance between these two phenotypes, particularly a deficit in the M2 phenotype, was identified in neuroinflammatory disorders like ischemic stroke, which was also observed in RIR injury recently [[47], [48], [49]]. Accordingly, the switching of microglia from the M1 to the M2 phenotype was viewed as a promising strategy for the management of RIR-related diseases [50]. Both in vitro or in vivo, rapamycin was capable of inhibiting microglial M1 polarization and favoring polarization towards the M2 phenotype by triggering autophagy, which in turn relieved glaucomatous injury [51]. Bioinformatics analyses also indicated that Sca-1^+^ derived exosomes exert their effects by suppressing M1 microglial polarization and subsequent inflammation, which may then attenuate ganglion cell apoptosis and preserve visual function following RIR injury [52].

RGCs are capable of projecting visual information to the visual cortex by generating action potentials, with the synapses of which are identified as the primary site suffering damage upon sustaining RIR injury [1,53]. It has also been reported that a reduction in the thickness of the IPL, where the dendrites of retinal ganglion cells make contact with the axons of interneurons in the retina, correlates with severe visual dysfunction [54,55]. In the present study, thinning of the retinal thickness and IPL layer, accompanied by the loss of cells in the GCL layer, was observed following the induction of an inflammatory response by RIR. GLP attenuated the aforementioned injuries while simultaneously reducing the number of TUNEL^+^ cells, which is regarded as another key indicator of neuronal damage within the retina. Surface markers are specific proteins or molecules expressed on the outer membrane of cells that are critical for the identification of cell types and the assessment of cell activation [56]. Microglia can therefore be distinguished based on specific markers: those associated with the M1 phenotype include CD86, INOS, and MHC-II, whereas those associated with the M2 phenotype are characterized by CD206, ARG-1, and Ym1 [57]. The combined use of PCR and IF showed that GLP downregulated the expression of CD86 and iNOS while elevating the level of CD206. Moreover, the quantified levels of inflammatory factors can be employed to ascertain the activity of microglial phenotypes [58], where elevating anti-inflammatory factors, such as IL-4, IL-10, and TGF-β, constitutes a crucial aspect of the management of inflammation in mid- and late-stages [59]. Quantitative analysis of the plasma revealed that GLP administration led to a reduction in the levels of IL-1β and TNF-α, accompanied by an elevation in the levels of IL-4 and IL-10, suggesting that GLP exerts anti-inflammatory effects within the retina after RIR.

Furthermore, the ratios of P-JAK2/JAK2 and P-STAT3/STAT3 were measured to elucidate the signaling events implicated in GLP-mediated microglial polarization. The JAK/STAT pathway, mainly activated by cytokines and their receptors, comprises two distinct protein families: JAKs and STATs [60,61]. Following the binding of extracellular ligands to the cytokine receptors, the JAKs are phosphorylated and activated, which results in the recruitment of the corresponding STATs proteins. The phosphorylated STATs subsequently translocate to the nucleus, where they regulate the transcription of specific genes, thereby exacerbating the neuroinflammation [62,63]. It is increasingly acknowledged that the activation of the JAK2/STAT3 signaling pathway is a crucial step in neuroinflammation mediated by microglia [[64], [65], [66]]. For instance, the aberrant activation of this pathway has been shown to induce chronic pain through its interaction with microglia, the release of pro-inflammatory cytokines, and the modulation of synaptic plasticity [64]. However, another study revealed that the efficacy of melatonin in alleviating neuroinflammation was blocked by the inhibitors of JAK2/STAT3, which suppressed the polarization of microglia from M1 to M2. The divergence in findings may be attributed to the interplay between JAK2 and telomerase [67]. Our findings indicated that GLP markedly lowered the phosphorylation levels of both JAK2 and STAT3, thereby inhibiting the activation of the STAT3-JAK2 signaling pathway in the retina.

To our knowledge, this is the first report to investigate the anti-inflammatory properties of GLP against RIR-induced retinal injury. The results suggest that GLP may modulate the microglial phenotype and suppress the neuroinflammatory responses, presumably mediated via the JAK2/STAT3 signaling pathway. Nevertheless, it was essential to acknowledge the limitations of the present study. To begin with, we verified a novel mechanism of the JAK2/STAT3 signaling pathway by which GLP attenuates microglia-mediated neuroinflammation after RIR. While other signaling pathways involved in this process, such as AKT/NFκB/NLRP3 and Nrf-2/HO-1, remain to be fully elucidated in future studies [68,69]. Another limitation is that the absence of clinical applications of GLP in the treatment of RIR-related disorders has made it necessary to accelerate the transition of the relevant basic research into clinical research in this field. Further evidence, both in vitro and in vivo, is required to substantiate the relationship between GLP and these mechanisms, which may be achieved by utilizing inhibitors or gene knockout rats.

## 5 Conclusion

In summary, our findings indicate that GLP exerts a neuroprotective impact against RIR-induced retinal injury by modulating the microglial phenotype and suppressing the neuroinflammatory responses, which are presumably mediated via the JAK2/STAT3 signaling pathway. These findings provide a novel insight into the mechanisms of GLP in ameliorating RIR and indicate that GLP may be viewed as a promising candidate for the management of RIR-related diseases.

## CRediT authorship contribution statement

**Guangyu Zhu:** Writing – review & editing, Writing – original draft, Methodology, Investigation, Conceptualization. **Yujie Liu:** Writing – review & editing, Writing – original draft, Formal analysis, Data curation. **Shichun Luo:** Writing – review & editing, Writing – original draft. **Chao Tang:** Writing – review & editing, Validation. **Chunlin Zhao:** Writing – review & editing, Visualization. **Xuejing Lu:** Supervision, Resources, Project administration, Methodology, Funding acquisition, Conceptualization.

## Ethical statement

The animal experiments were performed according to the welfare and ethical principles of laboratory animals approved by the Committee of laboratory Animal Welfare Ethics of Chengdu University of Traditional Chinese Medicine (SYXK2019-0049). The study was conducted using adult female SD rats, whose sex had no effect on the results of this experiment.

## Funding

This work was supported by the 10.13039/501100001809National Natural Science Foundation of China [grant number 82174444].

## Declaration of competing interest

The authors declare the following financial interests/personal relationships which may be considered as potential competing interestsXuejing LU reports financial support was provided by 10.13039/501100001809National Natural Science Foundation of China. If there are other authors, they declare that they have no known competing financial interests or personal relationships that could have appeared to influence the work reported in this paper.

## Figures and Tables

**figs1:**
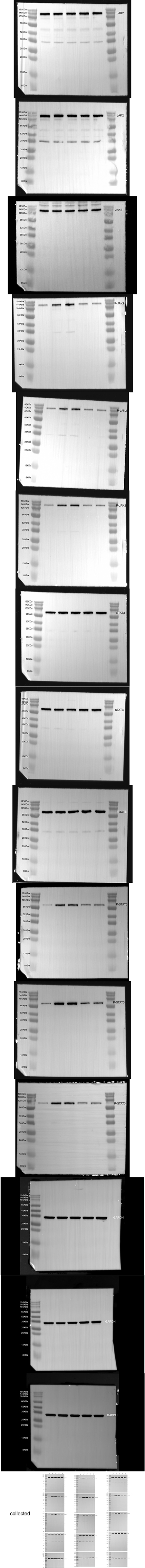


## Data Availability

Data will be made available on request.
